# Computational Approaches to Alkaline Anion-Exchange Membranes for Fuel Cell Applications

**DOI:** 10.3390/membranes12111051

**Published:** 2022-10-27

**Authors:** Cecil Naphtaly Moro Ouma, Kingsley Onyebuchi Obodo, Dmitri Bessarabov

**Affiliations:** HySA-Infrastructure, Faculty of Engineering, North-West University, Private Bag X6001, Potchefstroom 2520, South Africa

**Keywords:** alkaline anion-exchange membranes, microscopic, mesoscopic, macroscopic, computational approaches

## Abstract

Anion-exchange membranes (AEMs) are key components in relatively novel technologies such as alkaline exchange-based membrane fuel cells and AEM-based water electrolyzers. The application of AEMs in these processes is made possible in an alkaline environment, where hydroxide ions (OH^−^) play the role of charge carriers in the presence of an electrocatalyst and an AEM acts as an electrical insulator blocking the transport of electrons, thereby preventing circuit break. Thus, a good AEM would allow the selective transport of OH^−^ while preventing fuel (e.g., hydrogen, alcohol) crossover. These issues are the subjects of in-depth studies of AEMs—both experimental and theoretical studies—with particular emphasis on the ionic conductivity, ion exchange capacity, fuel crossover, durability, stability, and cell performance properties of AEMs. In this review article, the computational approaches used to investigate the properties of AEMs are discussed. The different modeling length scales are microscopic, mesoscopic, and macroscopic. The microscopic scale entails the ab initio and quantum mechanical modeling of alkaline AEMs. The mesoscopic scale entails using molecular dynamics simulations and other techniques to assess the alkaline electrolyte diffusion in AEMs, OH^−^ transport and chemical degradation in AEMs, ion exchange capacity of an AEM, as well as morphological microstructures. This review shows that computational approaches can be used to investigate different properties of AEMs and sheds light on how the different computational domains can be deployed to investigate AEM properties.

## 1. Introduction

**Figure 1:**
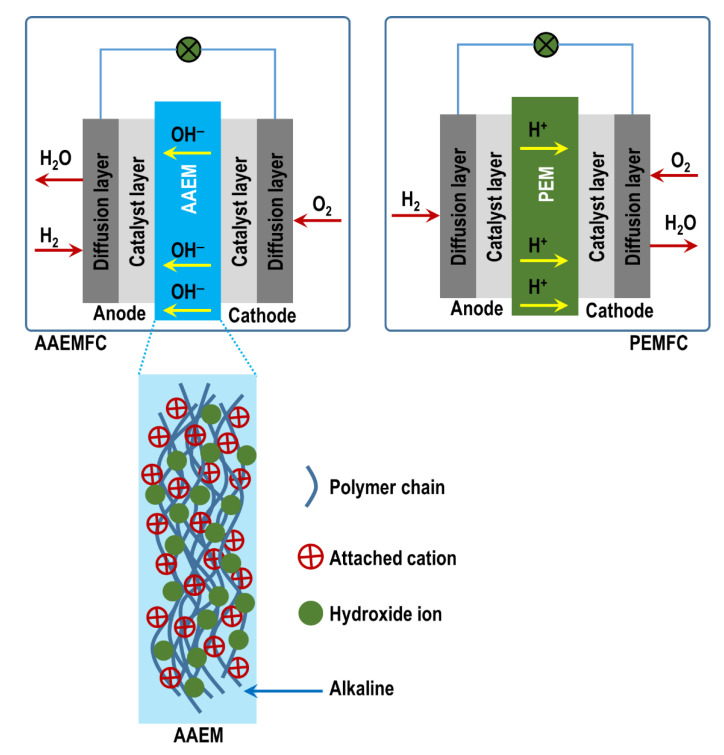
Schematic comparison between an AAEMMFC and a PEMFC.

When it comes to clean and efficient energy technologies for a sustainable future, fuel cells (FCs) are attracting increasing attention. To date, when it comes to research, development, and deployment, it is the low-temperature proton-exchange membrane fuel cells (PEMFC) that dominate, especially within the automotive sector [1,2]. However, alkaline anion-exchange membrane fuel cells (AAEMFCs) have been proposed as a possible challenger to PEMFCs—they have several functional attributes that make them direct competitors to PEMFCs [3]. Compared to PEMFCs, AAEMFCs can create opportunities for cost reduction, mainly due to the switch in operating conditions from an acidic to an alkaline environment/medium [4]. Alkaline media in AAEMFCs create an environment where inexpensive nonprecious metals [4] can be utilized as catalysts, in sharp contrast to PEMFCs which usually utilize expensive precious metals such as platinum-group metals as catalysts due to the acidic operating conditions. In addition, oxygen reduction under alkaline conditions in AAEMFCs is more effective compared to in PEMFCs under acidic conditions. An alkaline medium is also less corrosive and hence creates room for innovation. Figure 1 illustrates the difference between an AAEMFC and a PEMFC.

An AAEMFC also uses hydrogen as fuel, with oxygen as an oxidant. The reactions that take place at the electrodes are the following:

$$H_{2}{+2\mathrm{OH}}^{\text{–}}\;\to {\;2H}_{2}O+2e^{–}\;$$

at the anode,

$$O_{2}{+2H}_{2}O+4e^{–}\;\to \;4{\mathrm{OH}}^{–}$$

at the cathode, and the overall reaction in the AAEMFC is

$${2H}_{2}{+O}_{2}{+2\mathrm{OH}}^{\text{–}}\;\to {\;2H}_{2}O.$$

In AAEMFCs, the alkaline anion-exchange membrane (AAEM) is usually the key component that determines the performance of the AAEMFC [5,6]. It is for this reason that AAEMs have been/are being extensively investigated, both theoretically and experimentally. Investigations into catalysts for electrode (anode and cathode) applications have also been carried out

A good AEM must have high ionic conductivity, good mechanical, and thermal stability, and not be too costly. Thus, before AAEMFC technology can mature to the level of its PEMFC counterpart and gain a significant share of the FC power market, some issues need addressing, e.g., AAEM functional head groups, polymer structures, and membrane preparation methods.

Several excellent reviews exist in the literature on AAEMs and even AEMFCs [7,8,9,10,11]. Additionally, there are also reviews on the different modeling length scales as applied to the different applications [12]. This review succinctly focuses only on computational studies geared towards AAEM functional head groups, polymer structures, and alkaline electrolytes, among other topics—membrane preparation is not considered. Research into AAEMs stems from the need to develop suitable AAEMs for high-pH and high-temperature environments [1,12,13,14,15,16,17]. This review only focuses on AAEM application in AAEMFCs by zeroing in on how and where the computational techniques have been used and how they have informed research and development. Other reviews have aimed at giving a broad view of how computational techniques can be used to investigate AAEMs [12]. This review attempts to show that computational approaches have indeed been useful in studying the different properties of AAEMs. This review, however, only focuses on AEMs as applied to fuel cells and not AEMs as applied in electrolyzers. AEMs as applied in electrolyzers are beyond the scope of this work and hence were not considered.

The outline of this review is as follows: In Section 2 a brief overview of AAEMFCs is given; in Section 3 the different modeling length scales are summarized followed by how they have been applied to investigate the different properties of AAEMs for AAEMFC application; and Section 4 concludes.

## 2. Alkaline Anion-Exchange Membrane Fuel Cells (AAEMFCs)

The pivotal role of AAEMs is the transportation of hydroxide ions (OH^−^), produced at the cathode electrode, to the anode electrode. At the anode, the ions release electrons after undergoing a chemical reaction with the fuel. The AAEM is also responsible for inhibiting fuel crossover and the occurrence of a short circuit that acts as a selective barrier separating the anode and the cathode; and it also serves as an electrical insulator. Within an AAEM are polymer chains with positively charged functional groups; electroneutrality is maintained by mobile anions [18,19,20]. In an AAEM, it is the positively charged functional groups attached to the polymer backbone (see Figure 1) that are responsible for the mobility/diffusion of OH^−^ through the membrane. The following types of functionalized positively charged end groups have been commonly used in development efforts pertaining to AEM functional groups: quaternary ammonium, imidazolium, benzimidazolium, pyridinium, phosphonium, and pyrrolidinium [21].

**Figure 2:**
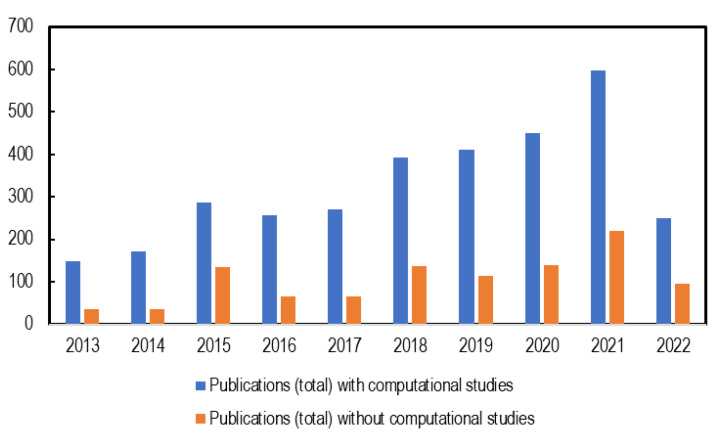
Number of AAEM publications per year (2013–2022, to date) with and without computational studies included.

Despite the numerous efforts geared towards the development of high-performance AAEMs, their application in AAEMFCs has been inhibited by the AAEMs’ low ionic conductivity and alkaline stability. Several studies have addressed these issues, experimentally, theoretically, or computationally [22,23,24]. There are instances where computational studies have been used to complement and/or augment experimental observations [24,25,26]; such studies have been used to either make predictions or to gain insights before commencing with experimental investigations. Over the past decade, there has been a noticeable increase in computational studies on AAEMs. As seen in Figure 2, computational techniques are contributing to studies investigating the properties of AAEMs even though experimental studies still dominate, hence the need to explore their capabilities and contributions.

## 3. Computational Studies of AAEMs

**Figure 3:**
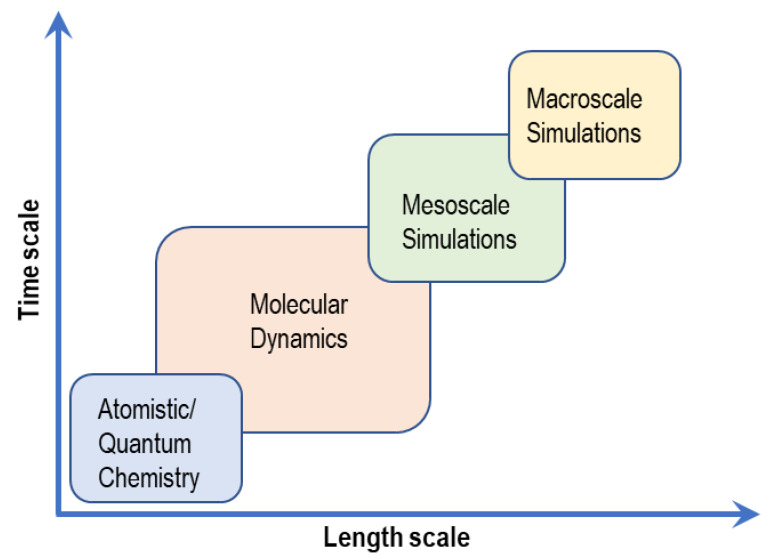
Computational modeling scales.

Arising from the need to understand how the properties of AAEMs can be improved, good use has been made of computational studies. Because of the diverse nature of computational techniques, they are often described or defined in terms of time and length scales. Some scales often overlap as they may require inputs from other scales, below or above. Figure 3 and Table 1 show the different computational techniques that can be used to model the properties of AAEM and AAEMFC components. Table 1 also shows and compares how the different modeling length scales have been used in previous studies to investigate AAEM properties.

### 3.1. Atomistic/Quantum Chemistry Studies of AAEMs

Atomistic and quantum chemistry studies employ quantum mechanical calculations to investigate the properties of AAEMs for AAEMFC applications. Density functional theory (DFT), in particular, has been used to investigate the properties of AAEMs and, in some cases, used to complement experimental investigations when it comes to gaining insight into the chemical stability of cationic polymers in alkaline media, as will be explained in the sections that follow below.

**Figure 4:**
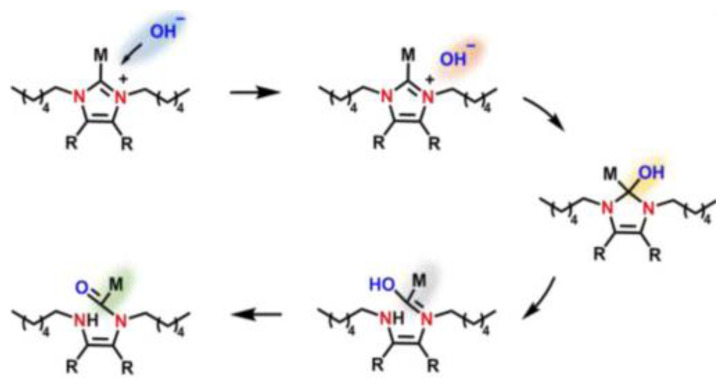
Schematic of degradation reaction process of a nucleophilic attack. Reprinted with permission from [24]. Copyright 2020 Elsevier.

DFT calculations have been used to investigate the chemical stability of N3-substituted (methyl, butyl, heptyl, dodecyl, isopropyl, and diphenyl methyl groups) imidazolium-based AAEMs that included both water and ethanol as solvents [26]. The DFT calculations were used to optimize the molecules as well as to understand the interaction between the lowest unoccupied molecular orbital (LUMO) and highest occupied molecular orbital (HOMO) energies of the cations and OH^−^ ions, respectively. The calculations revealed that the HOMO energies of the OH^−^ were lower than the LUMO energies of the imidazolium cation(s). This resulted in the cations inhibiting the nucleophilic attack from the OH^−^ (see Figure 4). The nucleophilic attack phenomenon is associated with LUMO energies being used to determine the alkaline stability of the imidazolium cations.

**Figure 5:**
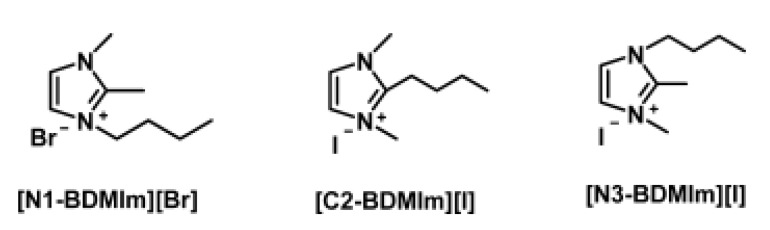
Molecular structures of imidazolium cations with different substitution positions. Reprinted with permission from [27]. Copyright 2014 American Chemical Society.

Transition state search calculations, also based on DFT, were used to confirm the nucleophilic attack—the most stable cation in alkaline solution at high temperatures obtained for DFT calculations was consistent with experimental observations in the same study. Si and coworkers [27] also investigated the alkaline stability of imidazolium cations experimentally and theoretically using DFT calculations (Figure 5). In their study, different substituents and substitution positions were considered, namely, imidazolium cations with butyl groups at various substitution positions (N1-, C2-, and N3-), 1-butyl-2,3-dimethylimidazolium ([N1-BDMIm]+), 2-butyl-1,3-dimethylimidazolium ([C2-BDMIm]+), and 3-butyl-1,2-dimethylimidazolium ([N3-BDMIm]+).

**Figure 6:**
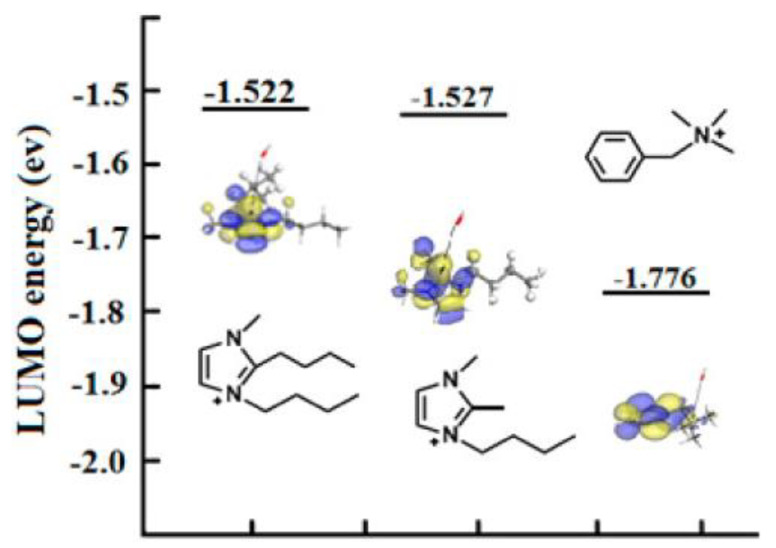
Isosurface and LUMO energy of the synthesized imidazolium cations. The color of atoms: red (O), blue (N), white (H), and grey (C). The black arrows indicate the nucleophilic attacking direction of OH^−^. Reprinted with permission from [27]. Copyright 2014 American Chemical Society.

Results of DFT calculations were consistent with experimental observations: C2 substitution (Figure 6) influenced alkaline stability, and there was high alkaline stability in the case of a butyl group-substituted imidazolium cation ([C2-BBMIm]+).

**Figure 7:**
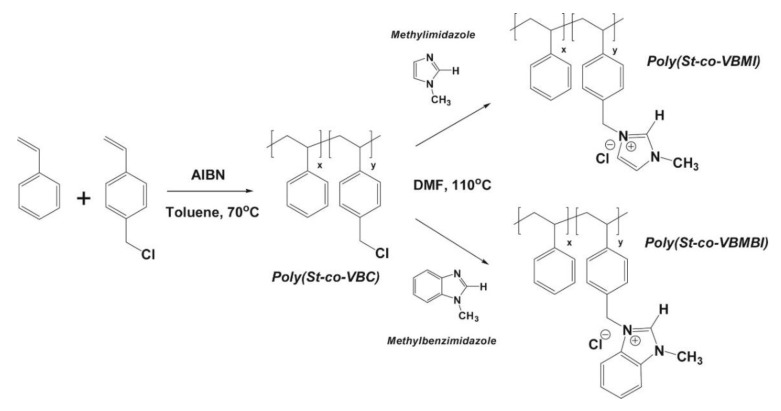
Molecules considered for 3-butyl-1-methylimidium (BMI) and 3-butyl-1-methylbenzimidium (BMBI). Reprinted with permission from [22]. Copyright 2017 Elsevier.

DFT calculations have also been used to investigate the nucleophilic attack/reaction in the stability of imidazolium- and benzimidazolium-based polyelectrolytes for AAEMs and also to confirm experimental results [22]. The molecules considered were 3-butyl-1-methylimidium (BMI) and 3-butyl-1-methylbenzimidium (BMBI) (see Figure 7).

**Figure 8:**
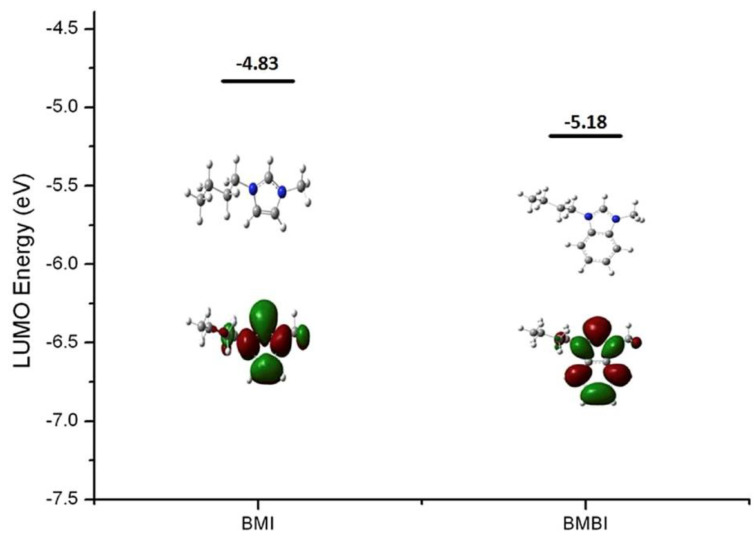
DFT-calculated LUMO energy and isosurfaces of model BMI and BMBI. (The grey, blue, and white balls represent C, N, and H atoms, respectively). Reprinted with permission from [22]. Copyright 2017 Elsevier.

It is evident from Figure 8 that BMBI has lower LUMO energy than BMI, indicating that BMBI will be susceptible to OH^−^ nucleophilic attack and hence will exhibit low or poor stability in alkaline media.

**Figure 9:**
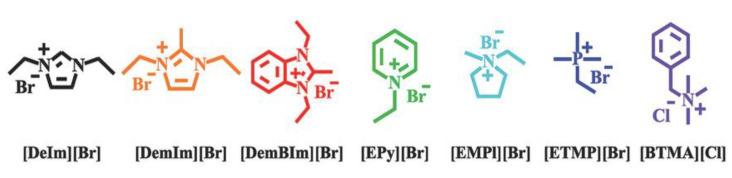
Molecular structures of cationic compounds investigated by Sun and coworkers. Reprinted with permission from [13]. licensed under CC BY-ND 4.0.

In another study [13], the alkaline stability of AAEMs in various alkaline media was investigated—alcohols (methanol, ethanol, propanol, and ethylene glycol) were used as fuel in the AAEMFCs. Here too, DFT calculations were used to obtain the LUMO energies of the cations, and, through this, cations with lower alkaline stability were identified. The results were consistent with experimental observations in the same study (Figure 9). The study also investigated the solvent effects with water, methanol, ethanol, and DMSO. In this case, DFT calculations revealed that the lower the dielectric constant of the solvent, the lower the alkaline stability of the cations. This was applied to explain the degradation of cations observed experimentally.

**Figure 10:**
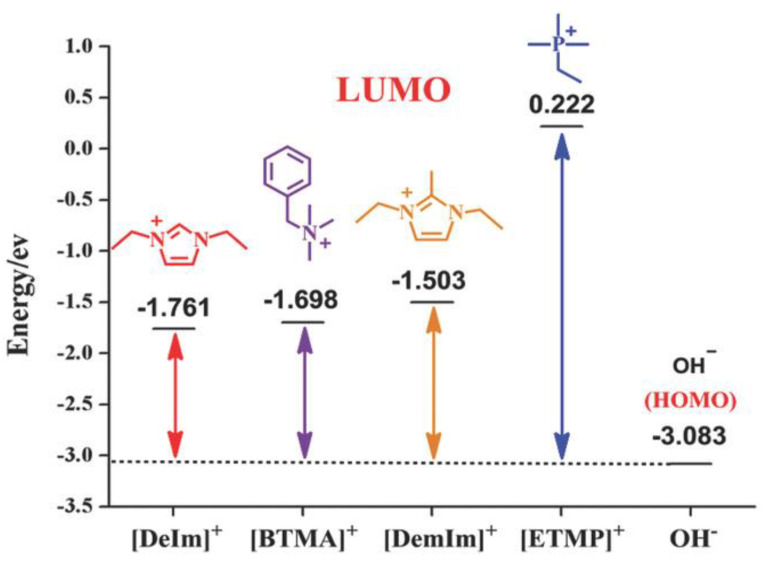
Frontier molecular orbital energy of various cations in water. Reprinted with permission from [13]. licensed under CC BY-ND 4.0.

Adsorption energies obtained from DFT calculations have also been used to explain catalyst deterioration [24], where achieving a decrease in adsorption energies of ionomers on the catalyst was suggested as a way of improving the performance of an AAEMFC. The same study also used DFT calculations to generate descriptors that were used to determine cation stability in alkaline media. A similar observation was made by Sun and coworkers[13] (see Figure 10) was also made by Maurya and coworkers [57] while investigating the interaction of the catalyst and the polymer electrolyte using DFT.

The chemical stability of the polymer backbones of AAEMs in alkaline media has also been investigated using DFT calculations [23]. Mohanty and coworkers, while investigating the chemical stability of different polymer backbones—using poly(arylene ether)s, poly(biphenyl alkylene)s, and polystyrene block copolymers as representative polymer structures—observed from DFT calculations that the enhanced long-term alkaline stability of AAEMs can be achieved using polymers without aryl ether bonds, i.e., all-carbon-based polymers. The reason offered is that electron-withdrawing groups near the aryl ether bonds accelerate the chemical degradation of the polymer backbone.

Recently, You and coworkers [28] used DFT calculations to complement their experimental study to investigate the alkaline degradation pathways of polymers. The DFT calculations in their study provided insight into why it is also important to consider the local backbone morphology of the polymer in the rational design of high-performance AAEMs that are chemically stable. This observation was made while using a one-pot, two-step process for synthesizing high-performance AAEMs [28]. In this instance, DFT offered insight into what was not previously known.

### 3.2. Molecular Dynamic Studies of AAEMs

DFT calculations, although fruitful in providing insight into and complimenting experimentally observed phenomena, are computationally expensive and cannot be used to investigate large systems or systems with many atoms. Calculations based on DFT are ground-state calculations in that they consider a system only in its ground state. Yet, most systems are never in their ground state. In cases where this is so, molecular dynamics (MD) calculations can be used. Even though MD calculations are not as accurate as DFT calculations when it comes to calculating some properties of a system, they are useful in providing insight into how a system dynamically evolves as a function of time, temperature, and pressure. MD calculations are computationally less expensive and thus can be used to investigate larger systems (systems larger than those that can be investigated using DFT calculations). When it comes to investigating the properties of AAEMs and AAEMFCs, MD calculations have also proven to be useful, and in many instances have been used to augment/compliment ab initio DFT calculations [29,35,36].

**Figure 11:**
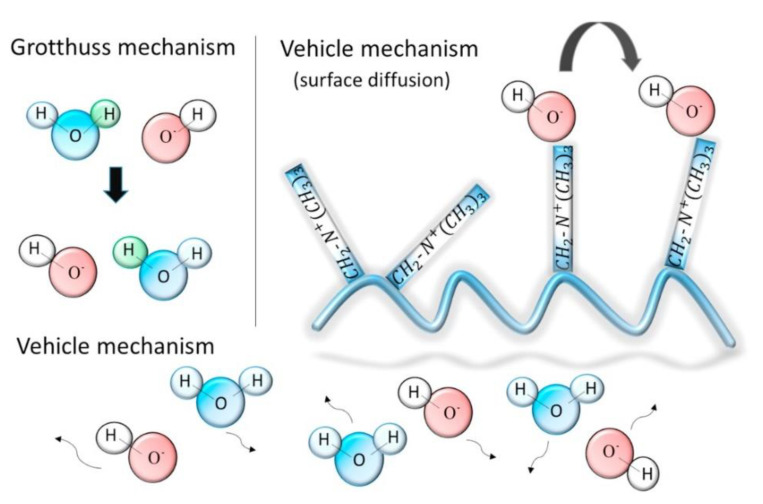
Hydroxide transport mechanisms in AAEMs. Reprinted with permission from [38]. Copyright 2017 Elsevier.

For AAEMFCs and AAEMs, experimental studies have not provided insight into the role of water when it comes to influencing ion diffusion, even though it has been implied that there exists a relationship between the water content and ionic conductivity [8,37,38]. The mobility of hydrated H^+^ and OH^−^ is usually high in alkaline media; thus, it was assumed that their mobilities are similar [38,57]. Some of the proposed ways through which OH^−^ is transported include the Grotthuss [8] and vehicle mechanisms [38,39]. These two OH^−^ transport mechanisms are illustrated in Figure 11.

**Figure 12:**
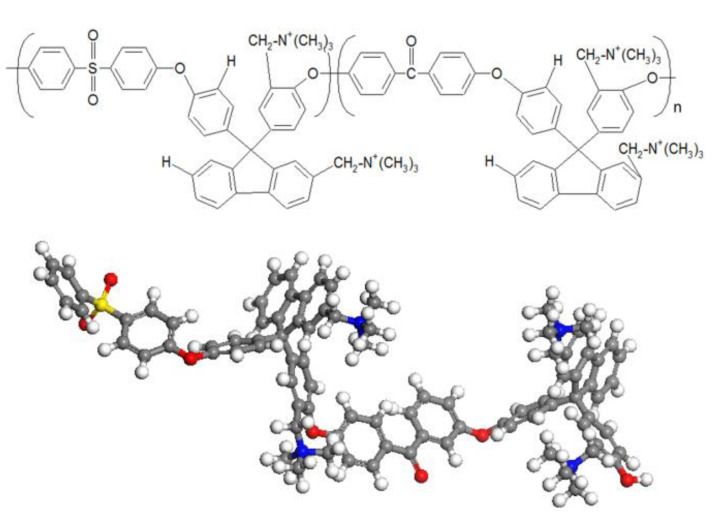
The QPE monomer used for classical molecular dynamics and first-principles molecular dynamics. Reprinted with permission from [38]. Copyright 2017 Elsevier.

Takaba and coworkers [38] employed MD calculations in the investigation of OH^−^ transport in AAEMs, particularly through poly(arylene ether sulfone ketone)s. They used two flavors of MD calculations, namely, classical MD and first-principles MD (FPMD), also commonly known as ab initio MD. The AAEM considered in the study contained quaternized ammonio-substituted fluorenyl groups (QPE) shown in Figure 12, and they used classical MD to understand how the OH^−^ ionic conductivity was impacted by the number of QPE repeat units. The observation made was that the conductivity of OH^−^ was not significantly influenced by increasing the number of QPE units. This observation was consistent with experimental observations.

**Figure 13:**
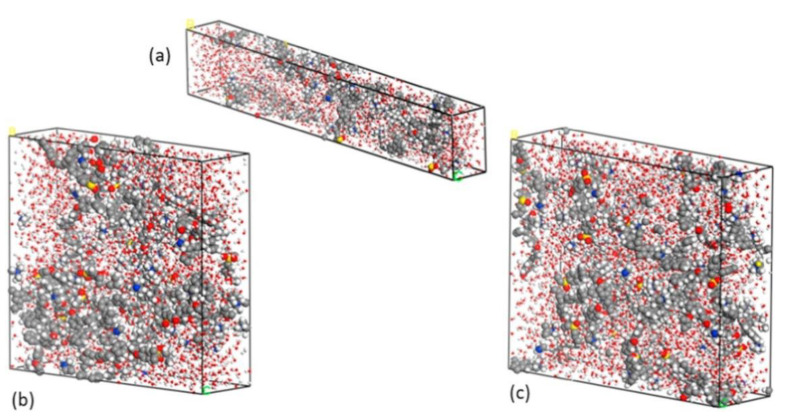
Images of (**a**) a unit cell wherein QPE comprises 10 repeating units, (**b**) a unit cell wherein QPE comprises 15 repeating units, and (**c**) a unit cell wherein QPE comprises 20 repeating units. Reprinted with permission from [39]. Copyright 2017 Elsevier.

They also used radial distribution functions (RDFs) obtained using classical MD to study the hydrated QPE and deduced the possibility of the existence of an interaction between the OH^−^ and ammonia groups—it was through this process that vehicle transportation was investigated. In the study, Grotthuss transport was investigated using force field (FF) MD, and the two transport mechanisms (Figure 11) showed that OH^−^ transport was similar to that of protons in a PEM. Figure 13 shows the size of the unit cells used for the MD calculations. Such systems would be computationally expensive if they were to be investigated using DFT calculations.

**Figure 14:**
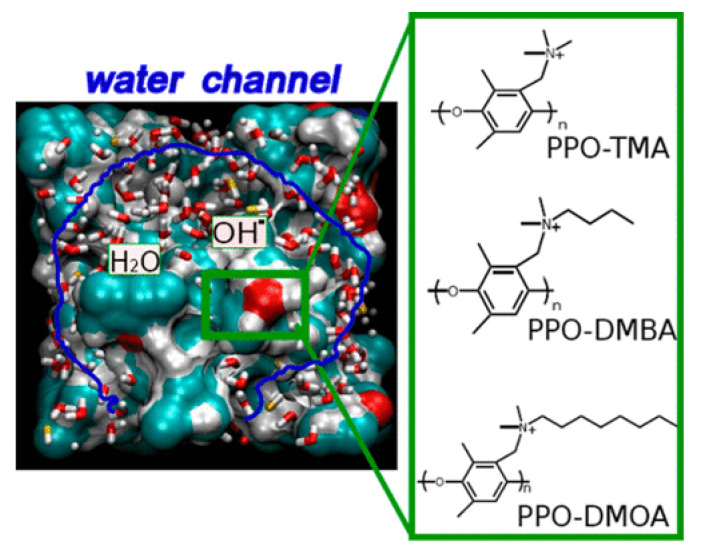
OH^−^ transport in hydrated AAEMs. Reprinted with permission from [41]. Copyright 2015 American Chemical Society.

A similar study was conducted by Wang and coworkers [40]. In their study, they considered the OH^−^ transport in polynorbornene AAEMs. Using RDFs calculated from the MD for amorphous cells of polynorbornene, they investigated the relationship between the polynorbornene microstructure and OH^−^ transport. Using ReaxFF reactive MD calculations, Zhang and van Duin [41] showed that OH^−^ diffusion improved with the hydration of AAEM microstructures in some cases, and thus it is considered important to balance membrane stability and conductivity. In their study, ReaxFF MD calculations were carried out on three functionalized poly(phenylene oxide) (PPO) AAEMs at two hydration levels (see Figure 14). The three AAEMs investigated were PPO–trimethylamine (PPO-TMA), PPO–dimethylbutylamine (PPO-DMBA), and PPO–dimethyloctylamine (PPO-DMOA). It was observed that the diffusion of OH^−^ increased with the swelling of the membrane microstructure affected by hydration because water molecules formed hydrophilic channels that mediated OH^−^ transport.

Enhanced OH^−^ conductivity in AAEMs has also been investigated in the case of monocationic and dicationic side chains, both experimentally and theoretically, using MD calculations. Several properties were investigated, including the alkaline stability and physiochemical and electrochemical properties [42]. In the case of dications, there was improved ionic conductivity and a better water uptake and swelling ratio of the membranes. Furthermore, the results of the MD calculations were consistent with experimental observations, namely, that the vehicular transport mechanism of OH^−^ was favored over the Grotthuss mechanism.

Chen and coworkers [30] investigated the effect of one (SQ) and two (GQ) quaternary ammonium-functionalized poly (ether ether ketone)/s on the ionic conductivity as well as the ion exchange capacity (IEC). Using coarse-grained MD calculations, they observed that improved ionic conductivity in GQ does not depend on the self-diffusion coefficients but rather on an increased IEC in GQ, and that the improved alkaline stability of GQ can be attributed to water molecules wrapping around the OH^−^. In another study of theirs [35], MD calculations were used to investigate the role of cationic groups in water sorption in AAEMs when it comes to steric hindrance and π-conjugation, in the case of trimethylammonium, 1-methylimidazolium, and tris(2,4,6-trimethoxyphenyl) phosphonium cations. It was reported that cations play a key role in ionic conductivity—both the steric effects and π-conjugation reduced cationic hydration, which, in turn, also reduced ionic conductivity.

**Figure 15:**
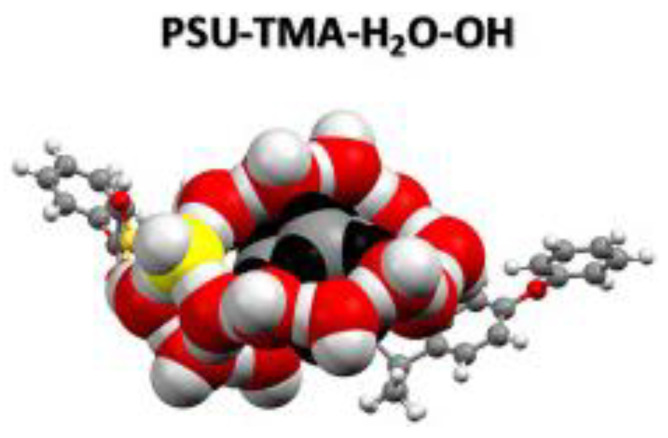
Optimized DFT structure used to predict the diffusion coefficients. Reprinted with permission from [29]. Copyright 2020 Elsevier.

DFT and MD calculations have been used to investigate how OH^−^ diffusion is influenced by water in fully swollen AAEMs as well as how the IEC influences the OH^−^ diffusion in AAEMs [29]. In the study of Di Salvo and coworkers, both modeling length scales, DFT and MD calculations, were used to complement each other (Figure 15). DFT was used to investigate the effect of membrane hydration and different IECs. The results were found to be consistent with experimental observations. In addition, DFT calculations were used to inform the boundary conditions of the MD calculations in that they were used to identify the critical IEC range where the membrane overswells. This range was subsequently used to obtain the OH^−^ diffusion coefficient using MD calculations, and the results were in good agreement with experimental observations.

Park and coworkers [31] used MD calculations to investigate the role of functional groups in channel morphologies for ionic conductivity as well as the chemical stability of AAEMs. Dubey and coworkers [32] employed empirical FF-based classical MD calculations to investigate and predict the solvation structure around the OH^−^ in the vehicular transport mechanism. This was done to utilize empirical FFs to overcome the limitations of ab initio calculations and the transferability of potentials while investigating OH^−^ transport in AAEMs. The chemical stability of cations and water in AAEMs has also been investigated using MD simulations [33]. Dekel and coworkers [33] observed a reduction in nucleophilicity due to the wrapping of water molecules around the OH^−^. In their study, experimental observations were used to corroborate the MD calculations. Yang and coworkers [34] while investigating poly(biphenyl N-methylpiperidine) (PBP)-based AAEMs have used both MD and DFT to complement their experimental study. In the study, the role of different quaternary ammonium cations on the backbone of PBP was investigated. The structure–activity relationship was investigated using MD calculations which showed that the performance of an AAEM depends on its structure. This is because the water channels that form in the microstructure of the PBP-based AAEM influence ionic conductivity and in turn the overall performance of an AAEMFC. In the same study, DFT calculations showed that the poor performance of PBP-based AAEMs emanated from the alkalinity (low basicity) as well as cation hydrophily.

### 3.3. Mesoscale Studies of AAEMs

Mesoscale simulations apply MD calculations in instances where the domains are too large when it comes to explicitly accounting for individual atoms and molecules in systems. It is akin to extending the applicability domain of MD simulations to explain the mesoscopic properties of a system. Mesoscale simulations have been used extensively to investigate polymer systems [43]. The most common technique used for mesoscale simulations of polymers is dissipative particle dynamics (DPD) [43]. The DPD mesoscopic simulation method has been used to investigate different mesoscopic structures of hydrated AAEMs [19,44,45,46]. Membrane degradation, as a result of the OH^−^ nucleophilic attack of cationic groups in AAEMs, is known to lead to a decrease in both the IEC and ionic conductivity [47,48]. Molecular simulations have been used to gain fundamental insights into AAEMs, with the aim of either accelerating experimental investigations or the rational design of high-performance AAEMs. As mentioned earlier, OH^−^ transport in AAEMs is influenced by several factors.

The morphology of hydrated AAEMs can be predicted using coarse-grained (CG) simulations at an affordable computational cost. However, because all the chemical interactions are lost in CG calculations, the key to the accuracy of CG simulations lies in FF construction [46]. Despite the application of DPD in modeling AAEMs, several issues have had to be addressed to make it applicable in terms of understanding the morphological properties of AAEMs. These include the inclusion of screening effects and electrostatic charges that are usually overlooked in DPD, as well as accurately representing the actual chemical species in the DPD force. DPD uses all-electron MD simulations to fit its model parameters. Of note, however, is the fact that all the modeling length scales have their merits. Atomistic calculations are plagued by computational costs, despite their accuracy in predicting properties, and FF construction plagues the accuracy of CG simulations even though they efficiently capture the morphology of the AAEMs [46].

**Figure 16:**
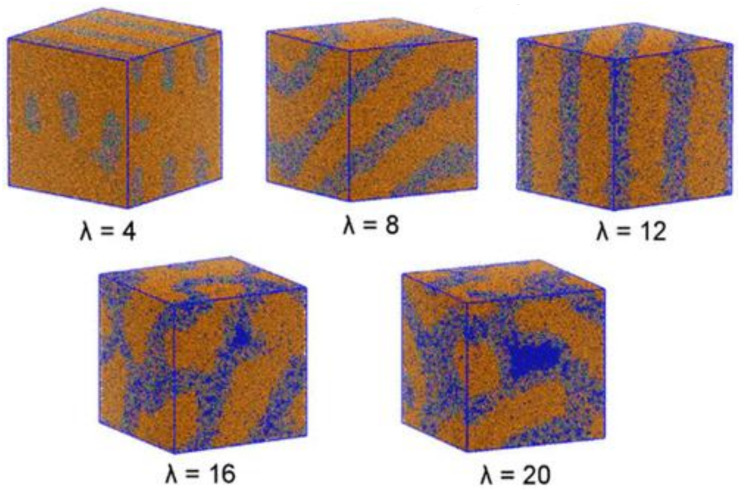
AAEM morphology simulated at different hydration levels. Reprinted with permission from [19]. Copyright 2017 American Chemical Society.

Sepehr and coworkers [19] investigated the hydrated morphology and microstructure of a triblock copolymer functionalized with alkyl-substituted quaternary ammonium groups in AAEMs using DPD. The copolymer was parametrized until the experimentally reported morphology was obtained. DPD simulations revealed that it was the degree of hydration that controlled the morphology of the AAEM copolymer. The morphology was observed to transform from perforated and interconnected lamellae to perfect lamellae to bicontinuous domains when the water content was varied from low to high (Figure 16). The observations made from the DPD calculations were consistent with experimental observations, particularly with regards to hydrophilic phase swelling upon hydration of the AAEM.

Lee [46] used DPD to investigate different side chains that could enhance the ionic conductivity; they looked at how hydration levels and IEC influence the mesoscale morphology of hydrated PPO-TMA, functionalized with tetramethylamine groups. In the study, the pathway for ion transport was expanded via the use of spacers tethered to PPO-TMA. It was observed from the mesoscale simulations that both the IEC and hydration levels played a direct role in the diffusivity of the water molecules as well as the anions. Furthermore, the addition of the spacers intensified phase segregation and the formation of water clusters, whose size depended on the amount of hydration as well as the spacers’ length. This study also offered insight into why there was retardation in the OH^−^ diffusivity due to the presence of water clusters.

In a follow up study, using mesoscale simulations, Lee [45] investigated the effect of alkyl side-chain modification on the ionic conductivity of PPO-TMA. In the study, a coarse-grain model of OH^−^ was used to track ionic conductivity as well as diffusion coefficients in situ as the calculation progressed. It was observed that ionic conductivity improved as a result of alkyl side-chain modification due to the hydrophobicity of the chains, which is required to mediate ion transport. Of note is that increased conductivity in the order of 26%–48% was observed. This was attributed to the microstructure of the polymer backbone being lamellar upon side-chain modification as well as the water channels that formed.

In yet another study, Lee [45] used mesoscale simulations to investigate the hydration of AAEMs with side-chain compositions of triblock copolymers. Aliphatic and aromatic polymer backbones, hydrophobic and hydrophilic spacers, and cation groups (single and multiple) were also considered in the study. Here, it was concluded that it is possible to improve the morphology of AAEMs and anion transport at high hydration levels through the use of hydrophilic spacers.

Luo and coworkers [49] investigated the mesoscale morphology of quaternary ammonium-tethered triblock copolymers. From mesoscale calculations, they observed that morphology was controlled by the degree of functionalization, the level of hydration, and the styrene content/percentage. Various meso-structure morphologies were observed when these parameters were varied: lamellar (perfect, crossed, and imperfect) and gyroid-like morphologies and the disordered, bi-continuous, and coexisting hollow micelles and layered structures.

The morphology of cation-functionalized AAEMs based on the triblock copolymer and polystyrene-b-poly(ethylene-co-butylene)-b-polystyrene have also been investigated using mesoscale DPD calculations [44]. Polystyrene and polyethylene functionalization was used to investigate hydrophilic and hydrophobic phases. In this study too, an increase in hydration levels resulted in morphology transformation from perforated and interconnected lamellae to perfect lamellae and eventually to a disordered bi-continuous morphology. An analysis of the calculated RDFs revealed that water distribution was influenced by the functional group, but the functional group had little effect on the polymer backbone.

### 3.4. Application of Machine Learning in Modeling Properties of AAEMs for AAEMFCs

Different modeling lengths and time scales have, of late, been used to generate descriptors for the rational design of novel materials. The descriptors have, in turn, been used to predict the properties of other novel materials when used as inputs for innovative modeling approaches such as training machine learning models. Machine learning (ML) coupled with artificial neural networks has been used to predict materials that would have otherwise been computationally intractable using DFT calculations. This is because a material’s configuration space is expansive to investigate using quantum mechanical calculations. Quantum mechanical calculations have been mostly used, due to their accuracy, when it comes to describing material properties.

The success of using DFT-calculated descriptors in the ML prediction of materials is evident in catalysts for the hydrogen evolution reaction (HER), for example. Experimental observations have also been used as descriptors in ML models, and, in some cases, descriptors obtained using DFT calculations and experiments have been used in the rational design of materials.

AAEMFCs have many complex components—including electrodes, polymers, and binding electrolytes, amongst others—all of which offer an opportunity for deploying ML models to investigate different systems. For example, there are several suitable cationic groups, and there are unlimited polymer configurations and ionomer conformations. There are also innovative approaches that can be used to improve particular properties of polymers (as addressed in the previous Section 3.1, Section 3.2 and Section 3.3). All these properties cannot be exhausted using the modeling approaches described. This then calls for the use of ML models in some cases. As was indicated in Figure 2, AAEM and AAEMFC research is a very active field—numerous publications have appeared annually over the past decade. All the information contained in these publications can be used to generate descriptors for use in ML models.

The use of ML has already been applied to PEMs using a database consisting of 789 data points that were obtained from only 30 publications (out of the many publications in the literature on the topic) [50]. This indicates that the deployment of ML models in this area of study is still in its infancy, but with great growth potential. Other ML studies have been carried out on different aspects of PEM electrolyzers [51,52,53]. The different aspects investigated are the HER [52] and oxygen evolution reaction catalysts as well as the anode porous transport layer (PTL) [51]. In the case of PTLs at the anode in polymer electrolyte water electrolyzers, 2000 images from X-ray computed tomography and CFD calculations were used to investigate oxygen transport using ML. Use was made of 18,000 configurations to investigate binary catalysts for cathode application in the case of the HER using ML [52]. DFT-generated descriptors for 15 transition metals were used to investigate anodic OER reactions using ML [53]. ML was also used to investigate transition metal dichalcogenides for HER applications using millions of datasets in a materials database [54].

**Figure 17:**
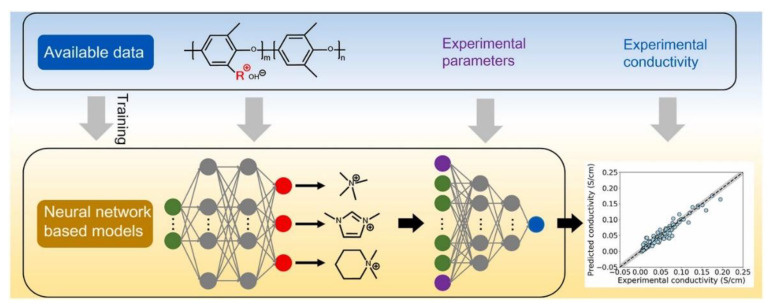
Machine learning methodology used to predict OH^−^ conductivity. Reprinted with permission from [55]. Copyright 2022 Elsevier.

AAEMs are also attracting attention when it comes to using ML to predict the OH^−^ ionic conductivity. The scheme that was followed by Zhai and coworkers[55] is presented in Figure 17. In their study, a deep learning algorithm was used to predict the OH^−^ conductivity of AAEMs from data extracted from published experimental studies. Keywords such as “anion exchange membrane” and “poly (2,6-dimethyl-1,4-phenylene oxide)/PPO” were used to source relevant publications from the Web of Science database. Only 64 papers were found that address PPO grafted with cationic groups.

**Figure 18:**
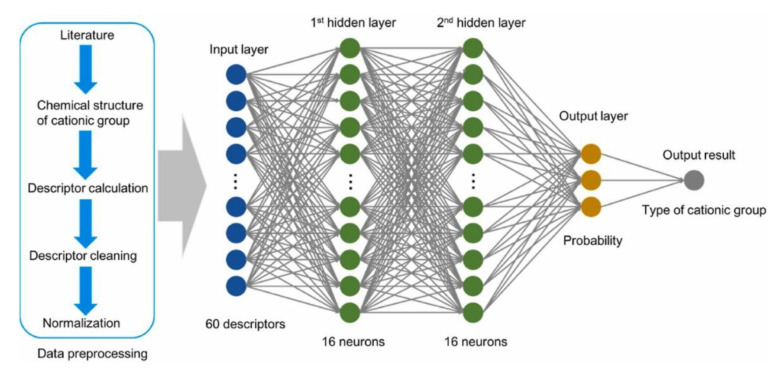
Classification model for identifying the type of functional cationic groups. Reprinted with permission from [55]. Copyright 2022 Elsevier.

The classification of cationic groups using the various descriptors extracted from the publication took place through the scheme shown in Figure 18.

Three prediction models were used to make OH^−^ conductivity predictions. These models were trained and tested. The trained models were then used to predict ionic conductivities for cationic groups that were not used in developing the models. However, the study did not go as far as considering polymer degradation.

Zou and coworkers [56] also used ML models, with five different algorithms and data from published literature between 2010 and 2020, to predict the chemical stability of AAEMs for AAEMFC applications. There are 86 publications that report experimental observations on conductivity retention. A database consisting of physical and chemical properties, membrane degradation, and the structure of polymers was constructed. Hammett substituent constants were used to quantify the chemical structures and the different AAEMs were classified using digital polymer structures. A decision tree was used to rank AAEMs with high alkaline stability, with the decision tree also suggesting spacers in the cases where unstable polymer backbones were introduced. Of the five ML models used, rapid and accurate predictions were obtained when an artificial neural network model was used. Thus, the use of ML in making predictions is likely to reduce the number of experiments that otherwise need to be carried out, to then better understand and design high-performance AAEMs.

### 3.5. Consistency of Modeling Length Scales with Experiments

As has been indicated in the respective Section 3.1, Section 3.2 and Section 3.3, computational techniques have been used to investigate the properties of AAEMs and have in some instances either offered insights on experimentally observed phenomena or have reported results consistent with experimental observations. Results from DFT calculations have been consistent with experimental observations as reported by [13,22,27,28] when it comes to alkaline stability in the case of a butyl group-substituted imidazolium cation [13,27], a nucleophilic attack/reaction [22], and the alkaline degradation pathways of polymers [28]. In the case of alkaline degradation pathways, DFT calculations provided insight into the importance of considering the local backbone morphology of the polymer in the rational design of high-performance AAEMs, which had not been revealed from experimental observations.

MD calculations have also been used to provide insights into the implied relationship between the water content and ionic conductivity [8,37,38]. Because the mobility of hydrated H^+^ and OH^−^ is high in alkaline media, their mobilities were assumed to be similar [38,57], with some experimental studies proposing that OH^−^ was transported through either the Grotthuss [8] or vehicle mechanisms [38,39]. The MD calculations revealed that the vehicular transport mechanism of OH^−^ was favored over the Grotthuss mechanism [38,42]. Consistency with experimental observations has also been observed in cases where MD calculations have been used to investigate the alkaline stability and physiochemical and electrochemical properties [42]; IEC influence on OH^−^ diffusion in AAEMs, membrane hydration, and different IECs [29]; the chemical stability of cations and water [33]; and the structure–activity relationship [34]. Mesoscale calculations have also been used to provide insights on the hydrated morphology and microstructure of a triblock copolymer functionalized with alkyl-substituted quaternary ammonium groups in AAEMs using DPD. DPD simulations revealed that it was the degree of hydration that controlled the morphology of the AAEM copolymer. This observation was consistent with experimental observations.

## 4. Conclusions

This review has addressed the capability of computational approaches in investigating the properties of AAEMs. In investigating different properties of AAEMs, the following topics have been discussed: different computational length scales that span quantum mechanical calculations using DFT; different flavors of the MD calculations; and mesoscale calculations. The application of different modeling length scales in AAEM studies is summarized in table form (Table 1). DFT calculations are limited by computational cost when it comes to investigating AAEM properties in large systems (systems with many atoms) despite being highly accurate in describing the membrane properties of AAEMs and AAEMFCs. The accuracy of MD calculations is sensitive to force field construction; thus, care should be taken in defining the actual interaction of the systems. DPD mesoscale calculations have been used to investigate the morphology of AAEM microstructure as a function of hydrogenation levels and IEC. ML has also been used to predict high-performance AAEMs as well as AAEM properties.

## Figures and Tables

**Table 1:** Application of different modeling length scales in AAEM studies.

Scale	Tools	Reference	Phenomena
Density functional theory (DFT)	DFT	[13,22,23,24,26,27,28]	Chemical stability, alkaline stability, polymers interactions, OH^–^ adsorption and diffusion, OH^–^ transport, nucleophilic attack, HOMO and LUMO energy
Molecular dynamics (MD)	Coarse-grained MD, first-principles MD, ab initio MD, classical MD, force fields, ReaxFF (reactive MD)	[8,29,30,31,32,33,34,35,36,37,38,39,40,41,42]	OH^–^ mobility and transport mechanism, cation head groups, polymer backbones, ionic conductivity, hydration (water uptake), ion exchange capacity (IEC)
Mesoscale simulations	Coarse-grained MD, dissipative particle dynamics	[19,43,44,45,46,47,48,49]	Alkaline stability, water uptake, IEC, hydrated morphology and microstructure, ionic conductivity
Machine learning		[50,51,52,53,54,55,56]	Alkaline stability, polymer configurations, catalysts, OH^–^ transport

## Data Availability

Data used in the study are included in the manuscript.

## References

[1] Sun Z., Lin B., Yan F. (2018). Anion-Exchange Membranes for Alkaline Fuel-Cell Applications: The Effects of Cations. Chem. Sus. Chem..

[2] Hickner M.A., Herring A.M., Coughlin E.B. (2013). Anion exchange membranes: Current status and moving forward. J. Polym. Sci. Part B Polym. Phys..

[3] Vincent I., Kruger A., Bessarabov D. (2017). Development of efficient membrane electrode assembly for low cost hydrogen production by anion exchange membrane electrolysis. Int. J. Hydrogen Energy.

[4] Gao X., He L., Yu H., Xie F., Yang Y., Shao Z. (2020). The non-precious metal ORR catalysts for the anion exchange membrane fuel cells application: A numerical simulation and experimental study. Int. J. Hydrogen Energy.

[5] Zhang F., Li T., Chen W., Yan X., Wu X., Jiang X., Zhang Y., Wang X., He G. (2020). High-Performance Anion Exchange Membranes with Para-Type Cations on Electron-Withdrawing C=O Links Free Backbone. Macromolecules.

[6] Tao Z., Wang C., Zhao X., Li J., Guiver M.D., Tao Z., Wang C., Li J., Zhao X., Guiver M.D. (2021). Progress in High-Performance Anion Exchange Membranes Based on the Design of Stable Cations for Alkaline Fuel Cells. Adv. Mater. Technol..

[7] Xiao F., Wang Y.C., Wu Z.P., Chen G., Yang F., Zhu S., Siddharth K., Kong Z., Lu A., Li J.C. (2021). Recent Advances in Electrocatalysts for Proton Exchange Membrane Fuel Cells and Alkaline Membrane Fuel Cells. Adv. Mater..

[8] Hren M., Božič M., Fakin D., Kleinschek K.S., Gorgieva S. (2021). Alkaline membrane fuel cells: Anion exchange membranes and fuels. Sustain. Energy Fuels.

[9] Sarapuu A., Kibena-Põldsepp E., Borghei M., Tammeveski K. (2018). Electrocatalysis of oxygen reduction on heteroatom-doped nanocarbons and transition metal-nitrogen-carbon catalysts for alkaline membrane fuel cells. J. Mater. Chem. A.

[10] Park E.J., Kim Y.S. (2018). Quaternized aryl ether-free polyaromatics for alkaline membrane fuel cells: Synthesis, properties, and performance-a topical review. J. Mater. Chem. A.

[11] Yang Y., Peltier C.R., Zeng R., Schimmenti R., Li Q., Huang X., Yan Z., Potsi G., Selhorst R., Lu X. (2022). Electrocatalysis in Alkaline Media and Alkaline Membrane-Based Energy Technologies. Chem. Rev..

[12] Karibayev M., Kalybekkyzy S., Wang Y., Mentbayeva A. (2022). Molecular Modeling in Anion Exchange Membrane Research: A Brief Review of Recent Applications. Molecules.

[13] Sun Z., Pan J., Guo J., Yan F. (2018). The Alkaline Stability of Anion Exchange Membrane for Fuel Cell Applications: The Effects of Alkaline Media. Adv. Sci..

[14] Mamlouk M., Manolova M. (2019). Chapter 6: Alkaline Anionic Exchange Membrane Water Electrolysers. Electrochemical Methods for Hydrogen Production.

[15] Henkensmeier D., Najibah M., Harms C., Žitka J., Hnát J., Bouzek K. (2021). Overview: State-of-the Art Commercial Membranes for Anion Exchange Membrane Water Electrolysis. J. Electrochem. Energy Convers. Storage.

[16] Vincent I., Bessarabov D. (2018). Low cost hydrogen production by anion exchange membrane electrolysis: A review. Renew. Sustain. Energy Rev..

[17] Du N., Roy C., Peach R., Turnbull M., Thiele S., Bock C. (2022). Anion-Exchange Membrane Water Electrolyzers. Chem. Rev..

[18] Hibbs 2022 M.R. (2013). Alkaline stability of poly(phenylene)-based anion exchange membranes with various cations. J. Polym. Sci. Part B Polym. Phys..

[19] Sepehr F., Liu H., Luo X., Bae C., Tuckerman M.E., Hickner M.A., Paddison S.J. (2017). Mesoscale Simulations of Anion Exchange Membranes Based on Quaternary Ammonium Tethered Triblock Copolymers. Macromolecules.

[20] Espiritu R., Tan J.L., Lim L.H., Arco S. (2020). Density functional theory study on the degradation of fuel cell anion exchange membranes via removal of vinylbenzyl quaternary ammonium head group. J. Phys. Org. Chem..

[21] Vijayakumar V., Nam S.Y. (2019). Recent advancements in applications of alkaline anion exchange membranes for polymer electrolyte fuel cells. J. Ind. Eng. Chem..

[22] Mohanty A.D., Tignor S.E., Krause J.A., Choe Y.K., Bae C. (2016). Systematic Alkaline Stability Study of Polymer Backbones for Anion Exchange Membrane Applications. Macromolecules.

[23] Pan J., Sun Z., Zhu H., Cao H., Wang B., Zhao J., Yan F. (2020). Synthesis and characterization of main-chain type polyimidazolium-based alkaline anion exchange membranes. J. Memb. Sci..

[24] Choe Y.K., Fujimoto C., Lee K.S., Dalton L.T., Ayers K., Henson N.J., Kim Y.S. (2014). Alkaline stability of benzyl trimethyl ammonium functionalized polyaromatics: A computational and experimental study. Chem. Mater..

[25] Gu F., Dong H., Li Y., Si Z., Yan F. (2014). Highly stable N3-substituted imidazolium-based alkaline anion exchange membranes: Experimental studies and theoretical calculations. Macromolecules.

[26] Si Z., Qiu L., Dong H., Gu F., Li Y., Yan F. (2014). Effects of substituents and substitution positions on alkaline stability of imidazolium cations and their corresponding anion-exchange membranes. ACS Appl. Mater. Interfaces.

[27] You W., Ganley J.M., Ernst B.G., Peltier C.R., Ko H.Y., DiStasio R.A., Knowles R.R., Coates G.W. (2021). Expeditious synthesis of aromatic-free piperidinium-functionalized polyethylene as alkaline anion exchange membranes. Chem. Sci..

[28] Luque Di Salvo J., De Luca G., Cipollina A., Micale G. (2020). Effect of ion exchange capacity and water uptake on hydroxide transport in PSU-TMA membranes: A DFT and molecular dynamics study. J. Memb. Sci..

[29] Chen S., Wang H., Zhang J., Lu S., Xiang Y. (2020). Effect of side chain on the electrochemical performance of poly (ether ether ketone) based anion-exchange membrane: A molecular dynamics study. J. Memb. Sci..

[30] Park C.H., Kim T.H., Kim D.J., Nam S.Y. (2017). Molecular dynamics simulation of the functional group effect in hydrocarbon anionic exchange membranes. Int. J. Hydrogen Energy.

[31] Dubey V., Maiti A., Daschakraborty S. (2020). Predicting the solvation structure and vehicular diffusion of hydroxide ion in an anion exchange membrane using nonreactive molecular dynamics simulation. Chem. Phys. Lett..

[32] Dekel D.R., Willdorf S., Ash U., Amar M., Pusara S., Dhara S., Srebnik S., Diesendruck C.E. (2018). The critical relation between chemical stability of cations and water in anion exchange membrane fuel cells environment. J. Power Sources.

[33] Yang L., Wang Z., Wang F., Wang Z., Zhu H. (2022). Poly(aryl piperidinium) anion exchange membranes with cationic extender sidechain for fuel cells. J. Memb. Sci..

[34] Chen W., Wu X., Li T., Yan X., Zhang Y., Wang X., Zhang F., Zhang S., He G. (2021). Structural contribution of cationic groups to water sorption in anion exchange membranes: A combined DFT and MD simulation study. Chem. Eng. Sci..

[35] Chen W., Fu Z., Wu X., Li T., Yan X., Wang X., Cui F., Zhang S., He G. (2022). Micro-phase separation promoted by electrostatic field in electrospinning of alkaline polymer electrolytes: DFT and MD simulations. Chem. Eng. Sci..

[36] Tanaka M., Koike M., Miyatake K., Watanabe M. (2010). Anion conductive aromatic ionomers containing fluorenyl groups. Macromolecules.

[37] Takaba H., Hisabe T., Shimizu T., Alam M.K. (2017). Molecular modeling of OH^−^ transport in poly(arylene ether sulfone ketone)s containing quaternized ammonio-substituted fluorenyl groups as anion exchange membranes. J. Memb. Sci..

[38] Grew K.N., Chiu W.K.S. (2010). A Dusty Fluid Model for Predicting Hydroxyl Anion Conductivity in Alkaline Anion Exchange Membranes. J. Electrochem. Soc..

[39] Wang C., Mo B., He Z., Xie X., Zhao C.X., Zhang L., Shao Q., Guo X., Wujcik E.K., Guo Z. (2018). Hydroxide ions transportation in polynorbornene anion exchange membrane. Polymer.

[40] Zhang W., Van Duin A.C.T. (2015). ReaxFF Reactive Molecular Dynamics Simulation of Functionalized Poly(phenylene oxide) Anion Exchange Membrane. J. Phys. Chem. C.

[41] Pan J., Zhu H., Cao H., Wang B., Zhao J., Sun Z., Yan F. (2021). Flexible cationic side chains for enhancing the hydroxide ion conductivity of olefinic-type copolymer-based anion exchange membranes: An experimental and theoretical study. J. Memb. Sci..

[42] Wang J., Han Y., Xu Z., Yang X., Ramakrishna S., Liu Y. (2021). Dissipative Particle Dynamics Simulation: A Review on Investigating Mesoscale Properties of Polymer Systems. Macromol. Mater. Eng..

[43] Zhu Z., Luo X., Paddison S.J. (2019). DPD simulations of anion exchange membranes functionalized with various cationic groups and associated anions. Solid State Ionics.

[44] Lee M.T. (2020). Designing Anion Exchange Membranes with Enhanced Hydroxide Ion Conductivity by Mesoscale Simulations. J. Phys. Chem. C.

[45] Lee M.T. (2019). Exploring Side-Chain Designs for Enhanced Ion Conductivity of Anion-Exchange Membranes by Mesoscale Simulations. J. Phys. Chem. C.

[46] Marino M.G., Kreuer K.D. (2015). Alkaline Stability of Quaternary Ammonium Cations for Alkaline Fuel Cell Membranes and Ionic Liquids. ChemSusChem.

[47] Edson J.B., Macomber C.S., Pivovar B.S., Boncella J.M. (2012). Hydroxide based decomposition pathways of alkyltrimethylammonium cations. J. Memb. Sci..

[48] Luo X., Liu H., Bae C., Tuckerman M.E., Hickner M.A., Paddison S.J. (2020). Mesoscale Simulations of Quaternary Ammonium-Tethered Triblock Copolymers: Effects of the Degree of Functionalization and Styrene Content. J. Phys. Chem. C.

[49] Cheng C., Wang B., Liu Z., Zhang G., Xie B., Tongsh C., Xi F., Chen W., Jiao K. (2022). Numerical investigation of design and operating parameter effects on permeability-differentiated alkaline fuel cell with metal foam flow field. Appl. Therm. Eng..

[50] Satjaritanun P., O’Brien M., Kulkarni D., Shimpalee S., Capuano C., Ayers K.E., Danilovic N., Parkinson D.Y., Zenyuk I.V. (2022). Observation of Preferential Pathways for Oxygen Removal through Porous Transport Layers of Polymer Electrolyte Water Electrolyzers. iScience.

[51] Jäger M.O.J., Ranawat Y.S., Canova F.F., Morooka E.V., Foster A.S. (2020). Efficient Machine-Learning-Aided Screening of Hydrogen Adsorption on Bimetallic Nanoclusters. ACS Comb. Sci..

[52] Wu L., Guo T., Li T. (2021). Machine learning-accelerated prediction of overpotential of oxygen evolution reaction of single-atom catalysts. iScience.

[53] Ran N., Sun B., Qiu W., Song E., Chen T., Liu J. (2021). Identifying Metallic Transition-Metal Dichalcogenides for Hydrogen Evolution through Multilevel High-Throughput Calculations and Machine Learning. J. Phys. Chem. Lett..

[54] Zhai F.-H., Zhan Q.-Q., Yang Y.-F., Ye N.-Y., Wan R.-Y., Wang J., Chen S., He R.-H. (2022). A deep learning protocol for analyzing and predicting ionic conductivity of anion exchange membranes. J. Memb. Sci..

[55] Zou X., Pan J., Sun Z., Wang B., Jin Z., Xu G., Yan F. (2021). Machine learning analysis and prediction models of alkaline anion exchange membranes for fuel cells. Energy Environ. Sci..

[56] Maurya S., Dumont J.H., Villarrubia C.N., Matanovic I., Li D., Kim Y.S., Noh S., Han J., Bae C., Miller H.A. (2018). Surface Adsorption Affects the Performance of Alkaline Anion-Exchange Membrane Fuel Cells. ACS Catal..

[57] Agmon N. (2000). Mechanism of hydroxide mobility. Chem. Phys. Lett..

